# Characterisation of HIV-1 transmission clusters and drug-resistant mutations in Denmark, 2004 to 2016

**DOI:** 10.2807/1560-7917.ES.2018.23.44.1700633

**Published:** 2018-11-01

**Authors:** Andreas Petersen, Susan A Cowan, Jens Nielsen, Thea K Fischer, Jannik Fonager

**Affiliations:** 1 Bacteria, Parasites and Fungi, Statens Serum Institut, Copenhagen, Denmark; 2 European Public Health Microbiology (EUPHEM) training programme, European Centre for Disease Prevention and Control (ECDC), Stockholm, Sweden; 3 Infectious Disease Epidemiology & Prevention, Statens Serum Institut, Copenhagen, Denmark; 4 Virus & Microbiological Special Diagnostics, Statens Serum Institut, Copenhagen, Denmark

**Keywords:** human immunodeficiency virus, HIV, antimicrobial resistance, sexually transmitted infections, epidemiology, surveillance

## Abstract

This study describes the prevalence of human immunodeficiency virus (HIV) drug resistance mutations among 1,815 patients in Denmark from 2004 to 2016 and characterises transmission clusters. POL sequences were analysed for subtype, drug resistance mutations and phylogenetic relationship. The prevalence of surveillance drug resistance mutations (SDRM) was 6.7%, while the prevalence of drug resistance mutations (DRM) with a clinical impact was 12.3%. We identified 197 transmission clusters with 706 patients. Patients 40 years or older were less likely to be members of a transmission cluster and patients in transmission clusters were less likely to be infected abroad. The proportion of late presenters (LP) was lower in active compared with inactive clusters. Large active clusters consisted of more men who have sex with men (MSM), had members more frequently infected in Denmark and contained a significantly lower proportion of LP and significantly fewer patients with DRM than small active clusters. Subtyping demonstrated that the Danish HIV epidemic is gradually becoming more composed of non-B subtypes/circulating recombinant forms. This study shows that active HIV-1 transmission has become increasingly MSM-dominated and that the recent increase in SDRM and DRM prevalence is not associated with more sustained transmission within identified transmission networks or clusters.

## Introduction

Despite the widespread use of highly active antiretroviral therapy (HAART) to suppress human immunodeficiency virus type 1 (HIV-1) replication, the emergence of HIV-1 drug resistance mutations (DRM) reduces the therapeutic options available and increases the risk of treatment failure and onward transmission of DRMs. From a public health perspective, transmitted drug resistance mutations (TDRM) are especially problematic because their prevalence can increase in the population and severely limit the therapeutic options available for treatment-naïve patients. Standard HAART treatment guidelines therefore recommend baseline genotypic testing in patients newly diagnosed with HIV to guide the choice of the first line of HAART. The prevalence of TDRM has been estimated at between 10% and 15% in Europe and North America but may be higher [1,2]. A recent study indicates a decreasing prevalence of TDRM in the United Kingdom (UK) [3]. A previous study on HIV-infected patients in Denmark between 2001 and 2009 identified a prevalence of TDRM of 6.1% [4].

In order to devise public health interventions aimed at reducing the spread of HIV, knowledge about both past and current characteristics of the HIV epidemic is crucial. Important information includes demographic characteristics such as sex, transmission mode, age, country of origin and country of infection of the patients as well as virus subtype/circulating recombinant form (CRF) composition and TDRM.

The aim of this study was to describe the prevalence of TDRM in Denmark during the period from 2004 to 2016 in order to evaluate whether TDRMs were present at higher frequency among specific groups of Danish HIV patients and to study any changes during the study period. In addition, we characterised transmission clusters with respect to changing trends, presentation status, DRMs, sex, age, transmission mode and country of origin and infection.

## Methods

### Study population

To monitor TDRM in Denmark, blood samples from newly diagnosed HIV patients, along with clinical and epidemiological information were sent to the National Virology Surveillance and Research Unit at Statens Serum Institut (SSI) in Copenhagen, Denmark for genotypic characterisation of the POL (Pr and RT) gene (SERO project) [4]. Inclusion criteria were a sample obtained no later than 6 months after the first positive HIV test conducted in Denmark, and no previous history of antiretroviral therapy. Further epidemiological data on the patients was supplied by the national surveillance of HIV in Denmark. The study period was 2004 to 2016.

### Ethical considerations

According to the Danish Act on Research Ethics Review of Health Research Projects, this study does not require approval by the ethics committees as it does not cause increased health risk or discomfort to patients. This was confirmed by the Committees on Health Research Ethics for the Region of Southern Denmark in a specific waiver of approval (VF20020258). Data were collected, stored and analysed as approved by the Danish data protection agency (J.nr. 2015–57–0102).

### Data analysis

POL sequences used for analysis in this study were generated as described previously [5]. The HIV subtypes were identified using the REGA HIV-1 Subtyping Tool - Version 3.0 [6]. Presentation status was assigned to patients in accordance with the consensus definition [7]: Patients with a CD4^+^ T-cell count below 350 cells/µL or with an AIDS-defining illness, regardless of CD4^+^ T-cell count, were classified as late presenters (LP). All others, those with a CD4^+^ T-cell count above 350 cells/µL and/or without any AIDS-defining illness, were designated as non-late presenters (NLP).

We assessed surveillance drug resistance mutations (SDRM) defined as such on the World Health Organization (WHO) 2009 SDRM list [8]. In addition, we analysed DRMs according to the mutations defined in the HIV drug resistance database (HIVdb version 8.2) [9]. Resistance levels and penalty scores (a measure of the effect of mutations on susceptibility) were recorded for all protease inhibitors (PI), nucleoside reverse transcriptase inhibitors (NRTI) and non-nucleoside reverse transcriptase inhibitors (NNRTI). Drug resistance interpretation was categorised in five levels: 1 = susceptible, 2 = potential low-level resistance, 3 = low-level resistance, 4 = intermediate resistance, and 5 = high-level resistance. Identified DRM in newly diagnosed HIV-1 patients in Denmark were described and stratified by sex, transmission mode, country of origin, country of infection and age at the time of sample collection. When several possible transmission modes were stated, we assigned them to one category in the following overriding order: men who have sex with men (MSM) over people who inject drugs (PWID) over heterosexual.

A maximum-likelihood phylogeny of aligned POL sequences was produced for each major subtype and CRF using MEGA7 [10]. The General Time Reversible model was used with bootstrapping (100 replicates). We used the resulting phylogeny to analyse transmission clusters with Cluster Picker [11]. Initial and main support threshold were set at 0.9 and the genetic distance threshold was set at 4.5. Transmission clusters were described in relation to transmission mode, age at time of sample, sex, country of origin, country of infection and DRM. Transmission clusters were defined as active if the newest patient in the cluster was sampled in 2015 or 2016. For analysis of cluster size, we defined clusters with 10 patients or more as large and clusters with less than 10 patients as small.

### Statistics

We used Poisson regression to describe factors contributing to the likelihood of being in a transmission cluster and the likelihood of having a DRM. From a multivariate model with all potential factors we determined factors of significant (p < 0.05) influence using backward selection. Fischer’s exact test was used for analysis of differences between proportions and Student’s t-test was used for analysis of differences between means. A two-tailed p value < 0.05 was considered significant.

## Results

### Study population

The study population consisted of 1,815 patients, which corresponds to ca 60% of all newly diagnosed HIV-infected people in Denmark in the study period. Men constituted 81% of the study population (Table 1). More than half of the patients were younger than 40 years when diagnosed. The most common transmission mode was sex between men (54%), followed by heterosexual contact (36%). The proportion of men who have sex with men (MSM) was higher in the last four years of the study period (59%; p = 0.01). Most patients originated from Denmark (63%), and most were infected in Denmark (58%, yearly variation between 49 and 68% through the study period).

**Table 1:** Demographic and epidemiological characteristics of newly diagnosed HIV-1 in Denmark, 2004–2016 (n = 1,815)

Characteristics	Study population (n = 1,815)		Patients in transmission cluster (n = 706)		Patients in active transmission cluster (n = 264)		Patients in transmission cluster with resistance mutation (n = 85)		Patients in active transmission cluster with resistance mutation (n = 36)	
	n	%	n	%	n	%	n	%	n	%
**Sex**										
Male	1,462	80.6	605	85.7	237	89.8	74	87.1	32	88.9
Female	351	19.3	100	14.2	27	10.2	11	12.9	4	11.1
Not reported	2	0.1	1	0.1	0	0	0	0	0	0
**Age**										
<30 years	426	23.5	189	26.8	81	30.7	23	27.1	10	27.8
30–39 years	610	33.6	244	34.6	89	33.7	32	37.6	16	44.4
40–49 years	453	25.0	165	23.4	55	20.8	16	18.8	3	8.3
50–59 years	220	12.1	79	11.2	28	10.6	10	11.8	4	11.1
≥60 years	106	5.8	29	4.1	11	4.2	4	4.7	3	8.3
**Transmission mode**										
Sex between men	986	54.3	449	63.6	189	71.6	57	67.1	22	61.1
Heterosexual sex	661	36.4	204	28.9	64	24.2	25	29.4	12	33.3
Injecting drug use	83	4.6	39	5.5	7	2.7	2	2.4	1	2.8
Blood transfusion	6	0.3	2	0.3	0	0	0	0	0	0
Other	9	0.5	1	0.1	0	0	0	0	0	0
Not reported/unknown	70	3.9	11	1.6	4	1.5	1	1.2	1	2.8
**Country of origin**										
Denmark	1,142	62.9	515	72.9	201	76.1	68	80	30	83.3
Other	650	35.8	183	25.9	58	22.0	17	20	6	16.7
Not reported	23	1.3	8	1.1	5	1.9	0	0	0	0
**Country of infection**										
Denmark	1,052	58.0	532	75.4	207	78.4	69	81.2	30	83.3
Other	629	34.7	138	19.5	49	18.6	12	14.1	5	13.9
Not reported/unknown	134	7.4	36	5.1	10	3.8	4	4.7	1	2.8

### HIV-1 subtyping

**Figure 1:**
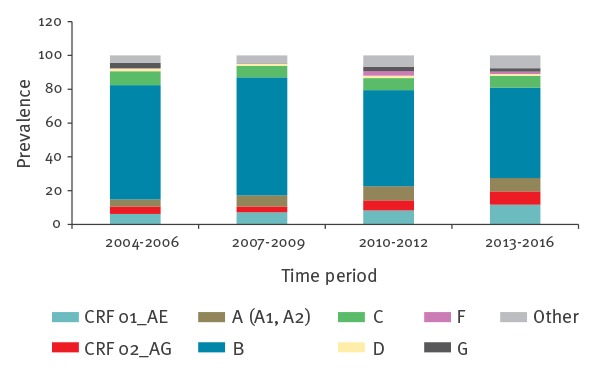
Distribution of HIV-1 subtypes and circulating recombinant forms in newly diagnosed patients in Denmark, stratified by time period, 2004–2016 (n = 1,815)

A total of 46 subtypes and CRFs were identified. Six samples could not be assigned to any previously described subtype. The most common types included B (61%) and CRF01_AE (8.6%, Table 2). The distribution of subtypes and CRFs varied throughout the study period. The prevalence of CRF 01_AE and CRF 02_AG increased from 6.2% to 12% (p = 0.005) and 4.4% to 7.7% (p = 0.04), respectively, from the beginning (2004–06) to the end (2013–16) (Figure 1). Similarly, subtype A (including A1 and A2) increased from 4.2% to 7.9% of the patients (p = 0.02). The prevalence of subtype B declined during the study period from 68% in the period 2004 to 2006 to 54% in the final years 2013 to 2016 (p = 0.0001; Figure 1). Non-B subtypes constituted 27% among men and 76% among women. The most prevalent non-B subtype among MSM was CRF01_AE (44/165; 27%). Nine of these patients originated from South-East Asia and among the 19 MSM with this subtype for whom the country of infection was known, eight reported Thailand.

**Table 2:** HIV subtypes and circulating recombinant forms among newly diagnosed HIV-1 patients in Denmark, 2004–2016 (n = 1,815)

Subtype/CRF	Study population (n = 1,815)		Patients in transmission cluster (any) (n = 706)		Patients in active transmission cluster (n = 264)		Patients in transmission cluster with resistance mutation (n = 85)		Patients in active transmission cluster with resistance mutation (n = 36)	
	n	%	n	%	n	%	n	%	n	%
B	1,113	61.3	560	79.3	217	82.2	67	78.8	24	66.7
CRF 01_AE	157	8.7	40	5.7	22	8.3	0	0	0	0
C	131	7.2	15	2.1	4	1.5	5	5.9	2	5.6
A (A1, A2)	125	6.9	32	4.5	3	1.1	2	2.4	0	0
CRF 02_AG	101	5.6	28	4.0	2	0.8	1	1.2	0	0
G	35	1.9	8	1.1	2	0.8	1	1.2	1	2.8
D	24	1.3	5	0.7	0	0	0	0	0	0
F (F1)	21	1.2	18	2.5	14	5.3	9	10.6	9	25
Other, including recombinants	102	5.6	0	0	0	0	0	0	0	0
Unassigned	6	0.3	0	0	0	0	0	0	0	0

CRF: circulating recombinant form.

CRF: circulating recombinant form.

### Resistance mutations

#### Surveillance drug resistance mutations

SDRM were demonstrated in 122 (6.7%) patients, 103 men and 19 women. Mutations conferring resistance to PI were found in 57 (3.1%) patients, mutations conferring resistance to NRTI were found in 41 (2.3%) patients, while 33 (1.8%) patients had mutations consistent with resistance to NNRTI. Three patients had dual mutations for PI and NRTI, and six patients had dual mutations for NRTI and NNRTI. These dual mutations were found in patients diagnosed throughout the study period. The prevalence of SDRM varied through the study period between 2.7% in 2012 and 8.2% in 2013 and 2014, with no clear trend. We analysed SDRM for subtype B vs non-B subtypes: For PI, resistance was 4.5% and 1.0% in B and non-B subtypes, respectively; for NRTI it was 2.9% and 1.4% and for NNRTI, it was 1.4% and 2.6%.

#### Clinically relevant drug resistance mutations

DRMs as defined by the HIVdb algorithm to at least level 2, potential low-level resistance, were identified in 223 (12%) patients, of whom 167 (66%) were male (Table 3, Figure 1). Four patients had PI and NRTI DRMs, three patients had PI and NNRTI DRMs and seven patients had NRTI and NNRTI DRMs. Diagnosis of patients with these dual mutations were evenly distributed throughout the study period. None of the epidemiological characteristics significantly predicted DRM in a Poisson regression model. DRM prevalence remained stable throughout the study period.

**Table 3:** Newly diagnosed HIV-1 infections with drug resistance mutations according to HIVdb level 2 (potential low-level resistance) or higher, Denmark, 2004–2016 (n = 223)

Drug class	All (n = 1,815)		In transmission cluster (n = 706)		In active transmission cluster (n = 264)	
	n	%	n	%	n	%
PI	52	2.9	15	2.1	5	1.9
NRTI	46	2.5	21	3.0	9	3.4
NNRTI	138	7.6	52	7.4	24	9.1
Any (PI, NRTI and/or NNRTI)	223	12.3	85	12.0	36	13.6

NNRTI: non-nucleoside reverse transcriptase inhibitors; NRTI: nucleoside reverse transcriptase inhibitors; PI: protease inhibitors.

The most frequently resistant sites in NNRTI between 2011 and 2016 were: E138 (32/77), V179 (19/77) and K103 (16/77). Ambiguous amino acid changes (polymorphisms) were observed in six cases for E138, in four for V179 and once for K103.

**Figure 2:**
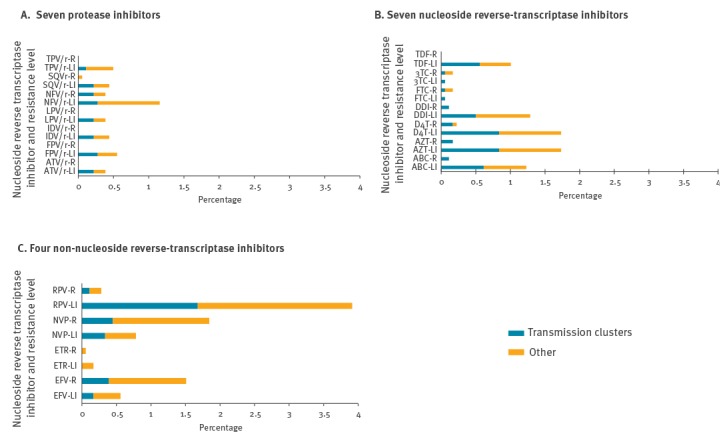
Prevalence of level of resistance (low-to-intermediate vs high-level) to different inhibitors, stratified by patients in and outside of (Other) transmission clusters (n = 1,815)

The prevalence of drug resistance levels 3–4 and 5 (low and intermediate combined, and high-level resistance), based on interpretation of the recorded mutations, is presented in Figure 2 and in the Supplement. For PI, one subtype had mutations resulting in high-level resistance to saquinavir/r (M46I and L90M) and seven subtypes had mutations leading to high-level resistance to nelfinavir (one M46I and L90M and six L90M). For NRTI, nine subtypes had different mutations leading to interpreted high-level resistance to abacavir (n = 2), zidovudine (n = 3), stavudine (n = 4), didanosine (n = 2), emtricitabine (n = 3) and lamivudine (n = 3), with some mutations leading to predicted resistance for several NRTI. For NNRTI, 32 of 1,815 subtypes (1.8%) had mutations interpreted as high-level resistance to nevirapine; 27 of these 32 subtypes resulted in high-level resistance to efavirenz and four of the same subtypes had interpreted high-level resistance to rilpivirine.

3tc: lamivudine; abc: abacavir; atv/r: atazanavir/r; azt: zidovudine; d4t: stavudine; ddi: didanosine; efv: efavirenz; etr: etravirine; fpv/r: fosamprenavir/r; ftc: emtricitabine; idv/r: indinavir/r; LI: low to intermediate resistance; lpv/r: lopinavir/r; nfv: nelfinavir; nvp: nevirapine; R: high-level resistance; rpv: rilpivirine; sqv/r: saquinavir/r; tdf: tenofovir; tpv: tipranavir/r.

### Identification of transmission clusters

We identified 197 transmission clusters containing 706 of the 1,815 patients (39%). During the study period, this fraction varied between 32% in 2004 and 44% in 2015, with no clear trend. Average cluster size was 3.6, with a minimum of two (111 clusters), and maximum of 27 (one cluster). Characteristics of the patients are presented in Table 1. Seventy-two clusters contained both men and women. Age group (p = 0.008) and country of infection (p < 0.001) were found to be the most important factors associated with being part of a transmission cluster. Patients aged 40 or older were less likely to be part of a transmission cluster and patients in transmission clusters were less likely to be infected abroad (Table 1). Prevalence of resistance (low and intermediate, and high-level) was similar within and outside of identified transmission clusters (Figure 2). A total of 49 clusters containing 264 patients were defined as active. Almost two thirds of those patients were younger than 40 years and the most common route of transmission was MSM (64%; Table 1). However, there was no significant difference between active and non-active transmission clusters for any of the characteristics. Of those reported to be infected outside Denmark, half (24/48) were Danish residents.

Subtypes B and F1 were more common in clusters than in non-clustered patients and more common in active than in inactive clusters (Table 2). Most other subtypes were present in lower proportions in transmission clusters compared with non-clustered patients.

DRM (HIVdb) were identified in 37 transmission clusters, most commonly NNRTI mutations (Table 3). The patients, 74 men and 11 women, mostly originated from and were infected in Denmark (Table 1). The prevalence of DRM in transmission clusters fluctuated and varied from 0% in 2016 to 23% in 2011, with no clear trend. Of the 37 transmission clusters with DRM, 13 clusters with 36 patients (32 men, four women) were defined as active. There was no statistically significantly difference in the prevalence of any DRM between active and inactive clusters. The most common NNRTI mutations E138, V179 and K103 were identified in, respectively, two subtype B clusters, one subtype F1 cluster and three active transmission clusters (two subtype B clusters and one subtype G cluster).

The average cluster size of active clusters was 5.4 compared with an average cluster size of 3.0 for inactive clusters. Seven of the 49 active clusters contained 10 or more patients, which was significantly more than the 148 older inactive clusters, among which only two contained 10 or more patients (Fischer’s exact test; p = 0.0009). The large active clusters consisted of more MSM, had members more frequently infected in Denmark, contained a significantly lower proportion of LP and consisted of significantly fewer patients with DRM compared with small active clusters (Table 4). In addition, subtype B was identified more frequently in the large than in the small active clusters (Table 4). Although patients in the large active clusters were on average slightly younger than patients in the smaller active clusters (36 vs 38 years), this was not statistically significant (Student’s t-test; p = 0.09).

**Table 4:** Characteristics of newly diagnosed HIV-1 patients and subtypes in active transmission clusters, stratified by cluster size, Denmark, 2004–2016 (n = 264)

Characteristic	In large active clusters (n = 120^a^)		In small active clusters (n = 144^a^)	
	n	%	n	%
Men who have sex with men	103	86^b^	84	60
Country of infection Denmark	107	92^b^	101	71
Late presenters	30	25	62	44^b^
Drug resistance mutations (HIVdb)	4	3.3	31	22^b^
Prevalence of subtype B	110	92^b^	77	53

^a^ The denominator is for some characteristics not equal to the number of patients in the clusters because of missing data.

^b^ Fischer’s exact test: p < 0.001.

### Presentation status

Presentation status could be assigned for 1,780 patients (98%), with 863 (48%) designated as NLP and 917 (52%) designated as LP. The proportion of LP fluctuated in the study period and was lowest in 2008 (42%) and highest in 2010 (60%). Presentation status had no significance in relation to being part of a transmission cluster, but active transmission clusters had fewer LP compared with inactive transmission clusters (incidence rate ratio (IRR) = 0.69: 95% confidence interval: 0.53–0.90).

In inactive and older transmission clusters, half of all DRM were identified among LP (26 LP with DRM among 50 with a known presentation status and a DRM in inactive clusters), whereas in active transmission clusters, this proportion was smaller, with 14 LP with DRM among 34 with a known presentation status and a DRM in active clusters. This difference in the proportion of DRM carried by LP was not statistically significant (p = 0.38), and although LP accounted for a higher proportion of DRM in active clusters (14/34) than the proportion of LP among all patients with a known presentation status in active clusters (92/264), that difference was also not statistically significant (p = 0.38).

## Discussion

In this study we report the prevalence of surveillance drug resistance mutations and clinically relevant resistant mutations in Denmark over a 13-year period. The average prevalence of SDRM was 6.7% over the entire period which did not differ much from a previous study comprising the years 2001 to 2009 [4]. The Danish situation resembles that in neighbouring Nordic countries and in Europe [1,12-14]. The current treatment recommendations in Denmark include two NRTI and as a third compound one of six drugs, of which only one (efavirenz) is an NNRTI [15], which may partly explain the low prevalence of SDRM to NNRTI (1.8%). No increase in the prevalence of PI, NRTI or NNRTI DRM was observed during the study period. This is in contrast to findings from a recent meta-analysis which found that the prevalence of NNRTI increased in most areas of the world, including Europe and North America [16]. In our study, NNRTI DRM was mainly caused by resistance mutations at three sites (E138, V179 and K103), which accounted for 82% of all NNRTI DRM in the study period and is comparable to what was recently reported in a study from Greece [17]. All three NNRTI DRM were identified in active transmission clusters composed of both B and non-B subtypes/CRF. The persistence of these NNRTI DRM in transmission clusters may, at least for K103N and E138A, be explained by the fact that these mutations have similar fitness as the wildtype [18,19].

The study as performed does not allow us to say whether the TDRM originated from patients who were drug-naïve or whether they originated from patients where therapy failed. A study from Switzerland found that treatment-naïve patients were a major source of TDRM [20]. Intensified screening to detect HIV infections earlier and allow early treatment would be necessary to stop such transmissions.

The WHO list for surveillance of DRM comprises mutations that provide clear evidence of drug exposure in a previous host and should therefore be indicators of TDRM. However, the list has not been updated since 2009 [8]. Several relevant resistance mutations are not included and some mutations included may have only limited clinical impact. Our results demonstrate that baseline mutation patterns should be assessed using the HIVdb algorithm to evaluate the clinical impact on drug susceptibility.

During the study period, the prevalence of subtype B decreased, while especially CRF01_AE, CRF02_AG and subtype A increased. This shows that the Danish HIV epidemic is gradually becoming more composed of non-B subtypes/CRFs, which is also a trend identified in other western European countries [12,21,22]. Several explanations of the phenomenon are possible: (i) increase in immigration and tourism from areas with non-B subtypes, (ii) travel-associated infections and (iii) increased internal circulation in Denmark of imported subtypes. The South-East Asian subtype CRF01_AE was the most common non-B subtype among MSM and was epidemiologically linked to this region in almost 40% of the cases. A similar trend was also observed in Switzerland [23]. Interestingly, while the prevalence of CRF02_AG increased in the overall population, its prevalence in active transmission clusters decreased. This suggests that while earlier clusters with CRF02_AG have been inactivated or remain dormant, its current high prevalence is mainly due to imported cases.

The results of this study are an indication of an ongoing endemic spread in Denmark. They highlight the importance of continued information campaigns among MSM and other persons at risk of becoming HIV-positive about getting tested regularly in order to be put on immediate HAART treatment and thus become unable to transmit the virus. Transmission in clusters was found to involve younger people. Social networking mobile apps such as Grindr, which facilitates anonymous contacts between gay and bisexual men, may contribute to this phenomenon.

Our results demonstrate two trends: (i) MSM with high transmission rates and a short time between infection and diagnosis and (ii) heterosexual people with low transmission rates and longer time from infection to diagnosis as well as a higher prevalence of DRM. These trends have been described earlier [24] and in addition, the present analysis shows that identifiable transmission networks are increasingly composed of large MSM-dominated clusters.

The patients of non-Danish origin in active transmission clusters could either be patients infected outside Denmark and become source cases in transmission clusters in Denmark, or they could be immigrant couples infected in home countries before entry in Denmark. The 24 patients of foreign origin and infected outside Denmark were distributed in 18 different transmission clusters, supporting the first explanation and thus demonstrating that HIV is also being imported and transmitted into endemic Danish clusters. Also, it is conceivable that some of the patients in our study were part of larger European clusters.

Interestingly, we observed that the proportion of LP in active clusters was lower than in inactive clusters. This could, at least partly, be explained by the observation that larger MSM-dominated clusters with a low proportion of LP increasingly dominate the epidemic. In Denmark a large effort has been put into getting MSM to test for HIV regularly and frequently. This has been a success as their median CD4^+^ T-cell numbers are substantially higher than heterosexuals at time of diagnosis [25]. LP in both active and inactive clusters carried slightly more DRM than expected from their proportion in the clusters, although this was not statistically significant. Although TDRM have been found to occur frequently among LP [26], it is not clear whether TDRM tend to be over- or underestimated during the prolonged absence of HAART in LP. Another potential explanation could be that LP at some point have already received treatment elsewhere, which was not reported. Future studies, using more sensitive sequencing technology should determine if LP harbour more TDRM than detected by standard Sanger sequencing.

We used a consensus definition of LP, relying on CD4^+^ T-cell count and AIDS-defining symptoms [7]. It has been argued that the consensus definition overestimates the proportion of LP [27]. The authors proposed using clinical information, including a recent negative test, a typical clinical presentation of acute infection and a history of recent risk behaviour with a known HIV-positive partner. The primary dataset in our study did not include the required information for all cases, e.g. date of negative HIV test was only available for ca 50%. In order to analyse the full dataset, we therefore used the consensus definition because we had CD4^+^ T-cell counts for 98% of our study material. A recent study has shown that infection time estimates derived from ambiguous nucleotide calls based on next generation sequencing (NGS) is more accurate than Sanger-based ambiguous nucleotide calls or other biomarkers [28]. Thus, NGS seems to be the state of the art with regards to HIV infection biomarkers. However, it was not feasible for this study to generate NGS data from all of the 1,815 samples in our study.

In this study, 60% of the patients were not members of a transmission cluster. According to their own information, most of them were infected in Denmark and it should therefore be possible to establish a link through phylogenetic analysis. However, it is estimated that 10% of a total number of 6,000 HIV-positive persons in Denmark are undiagnosed. Furthermore, this study represents only ca 60% of all newly diagnosed HIV patients in Denmark during the study period because of the inclusion and exclusion criteria in the SERO project. This could have prevented us from detecting epidemiological links through the phylogenetic analyses and thus, the number of patients in transmission clusters is most probably higher than reported here.

Identification of transmission clusters relies on the parameter genetic distance threshold in Cluster Picker. In this study we used 4.5. Using a smaller threshold of e.g. 1.5 could reduce long-lived transmission clusters into several smaller, independent and possibly active subclusters [29]. We used 4.5 to identify such long-lived clusters and then defined active clusters by patients having a first positive sample in the last 2 years of the study period.

Another limitation in this study is the quality of the epidemiological information. Upon inclusion in the SERO project, a dedicated nurse fills in a questionnaire on epidemiological risk factors with data provided by the patient. The information is therefore based on the self-reported answers. The question on country of infection may be difficult to answer correctly if patients have been sexually active both in Denmark and abroad before a positive HIV test.

A third possible limitation of the study is that men were slightly overrepresented. During the study period, the proportion of men among all newly diagnosed persons was 74%, compared with the 81% in this study, while the age distribution among all patients in the study was very similar to the overall age distribution in Denmark [25]. We do not think this small difference has any influence on our overall results.

## Conclusion

In summary, this study has both confirmed the persistence of earlier reported trends in the Danish HIV epidemic and shown that active HIV-1 transmission has become increasingly MSM-dominated. It has also shown that the recent increase in SDRM/DRM prevalence is not associated with more sustained transmission within identified transmission networks/clusters. The analysis presented in this study could only be performed by combining epidemiological and molecular data. Molecular data allow for a continued surveillance of TDRM at baseline and, from a public health perspective, information about transmission clusters with high transmission rates and/or high prevalence of TDRM allows for targeted interventions. It has also been suggested that the increased use of Pre-exposure prophylaxis among people at risk for HIV infection makes it even more important to continuously monitor TDRM and transmission trends to ensure the sustainability of this intervention method [30]. In order to better inform and maintain treatment regimens, the European Centre for Disease Prevention and Control is currently evaluating the capacity for HIV molecular surveillance within the European Union and European Economic Area [31,32].

## Supplement

144000 Supplement
