# Supplementary material for: Characterisation of HIV-1 transmission clusters and drug-resistant mutations in Denmark, 2004 to 2016
Source: Euro Surveill. 2018 Nov 1;23(44):1700633. doi: 10.2807/1560-7917.ES.2018.23.44.1700633 (PMC6337072; doi:10.2807/1560-7917.ES.2018.23.44.1700633)
Supplement: Supplement [file 10.28071560-7917.ES.2018.23.44.1700633-supp1.pdf]

Supplementary Table

Number of specific amino acid changes in the POL gene leading to low, intermediate or high resistance (levels 3-5) to protease inhibitors (PI), nucleoside reverse transcriptase inhibitors (NRTI) and non-nucleoside reverse transcriptase inhibitors (NNRTI) as assessed by HIVdb. Denmark 2004-2016.

This supplementary material is hosted by Eurosurveillance as supporting information alongside the article [Characterisation of HIV-1 transmission clusters and drug-resistant mutations in Denmark, 2004 to 2016] on behalf of the authors who remain responsible for the accuracy and appropriateness of the content. The same standards for ethics, copyright, attributions and permissions as for the article apply. Eurosurveillance is not responsible for the maintenance of any links or email addresses provided therein.

Drug class	Amino acid substitution	Number
PI		
PR major	M46I	1
	M46L	6
	M46MI	2
	L90M	6
	M46I,L90M	1
PR Accessory	L10LF	3
	K20T	1
	K20KT	4
	K20KIRT	1
	M46MV	1
	F53L	1
	Q58E	6
	Q58QE	2
	G73GA	1
	N83NDH	1
NRTI	M41L,L210LW	1
	M41L,L210W,T215S	2
	M41L,T215D	5
	M41L,T215E	2
	M41L,T215S	2
	D67N,K219E	1
	D67N,T215C,K219Q	1
	D67N,T69D,K70R	2
	D67N,T69D,T215FLPS	1
	T69D	1
	T69DN	1
	K70KN	1
	L74I	1
	Y115YF	1
	Q151M,T215S	1
	Q151QL	2
	M184I	1
	M184MV	1
	M184V	1
NRTI (continued)	L210W,T215E	1
	T215A	1
	T215D	4
	T215DGNS	1
	T215E	2
	T215S	3
	T215TA	1
	T215Y	1
NNRTI	A98G	4
	K103KN	2
	K103N	15
	K103N,E138A	1
	K103N,P225H	1
	K103N,Y188F	3
	K103S	1
	V106VM	1
	V108I	3
	V108I,E138A	1
	V108VI	3
	E138A	40
	E138A,V179E	1
	E138EA	6
	E138EG	1
	E138EK	1
	E138G	2
	E138K	3
	V179VL	1
	Y181C	1
	Y181I	1
	Y188L	3
	G190A	3
	G190GE	1
	H221HY	1
	H221Y	1
	Y318YF	1
